# Neurofascin and Compact Myelin Antigen-Specific T Cell Response Pattern in Chronic Inflammatory Demyelinating Polyneuropathy Subtypes

**DOI:** 10.3389/fneur.2018.00171

**Published:** 2018-03-19

**Authors:** Jan-Markus Diederich, Maximilian Staudt, Christian Meisel, Katrin Hahn, Edgar Meinl, Andreas Meisel, Juliane Klehmet

**Affiliations:** ^1^Neurocure Research Center Berlin, Charité University Medicine, Berlin, Germany; ^2^Department of Medical Immunology, Charité University Medicine, Berlin, Germany; ^3^Department of Neurology, Charité University Medicine, Berlin, Germany; ^4^Clinical Neuroimmunology, Ludwigs-Maximilians University, Munich, Germany

**Keywords:** chronic inflammatory demyelinating polyneuropathy, neurofascin, myelin basic protein, myelin protein zero, T cell response, chronic inflammatory demyelinating polyneuropathy subtypes

## Abstract

**Objective:**

The objective of this study is to investigate whether chronic inflammatory demyelinating polyneuropathy (CIDP) and its subtypes differ in their type 1 T-helper (TH1) cell response against nodal/paranodal neurofascin (NF186, NF155) as well as myelin protein zero (P0 180–199) and myelin basic protein (MBP 82–100).

**Methods:**

Interferon-gamma (IFN-γ) enzyme-linked immunospot assay was used to detect antigen-specific T cell responses in 48 patients suffering typical CIDP (*n* = 18), distal acquired demyelinating polyneuropathy (*n* = 8), multifocal acquired demyelinating sensory and motor polyneuropathy (MADSAM; *n* = 9), and sensory CIDP (*n* = 13) compared to other non-immune polyneuropathy (ON; *n* = 19) and healthy controls (*n* = 9).

**Results:**

Compared to controls, MADSAM and sensory CIDP patients showed broadest IFN-γ T cell responses to all four antigens. Positive IFN-γ responses against two or more antigens were highly predictive for CIDP (positive predictive value = 0.95) and were found in 77% of CIDP patients. Patients with limited antigen-specific response were females, more severely affected with neuropathic pain and proximal paresis. The area under the receiver operating characteristics curve (AUC) of NF186 in MADSAM was 0.94 [95% confidential interval (CI) 0.82–1.00] compared to ON. For sensory CIDP, AUC of P0 180–199 was 0.94 (95% CI 0.86–1.00) and for MBP 82–100 0.95 (95% CI 0.88–1.00) compared to ON.

**Conclusion:**

Cell-mediated immune responses to (para)nodal and myelin-derived antigens are common in CIDP. TH1 response against NF186 may be used as a biomarker for MADSAM and TH1 responses against P0 180–199 and MBP 82–100 as biomarkers for sensory CIDP. Larger multicenter studies study are warranted in order to establish these immunological markers as a diagnostic tools.

## Introduction

Chronic inflammatory demyelinating polyneuropathy (CIDP) is a rare autoimmune disorder of the peripheral nervous system and can be divided clinically into typical CIDP and atypical variants, such as distal acquired demyelinating polyneuropathy (DADS), multifocal acquired demyelinating sensory and motor polyneuropathy (MADSAM), and sensory CIDP (1, 2). Due to its heterogeneous manifestation, different autoimmune targets are likely to be relevant in CIDP (3). T cell responses have been shown to be involved in the immunopathogenesis of CIDP (4, 5). Previously, we and others detected autoreactive T cell responses against the compact myelin antigenic epitopes P2, PMP-22 as well as myelin protein zero 180–199 (P0 180–199), myelin basic protein 82–100 (MBP 82–100) measured by enzyme-linked immunospot (ELISPOT) assay (6–8). Additionally, we found differences between typical and atypical CIDP in antigenic response against P0 180–199 and MBP 82–100 (8).

There is an emerging body of evidence that molecules of the nodal/paranodal complex may be essential targets for blocking propagation of nerve impulses along myelin fibers (9). Thus, autoantibodies against the paranodal protein neurofascin 155 (NF155) have been identified in CIDP patients as well as patients suffering from combined central and peripheral demyelination (10, 11). Antibodies against the nodal NF186 have also been found in CIDP (12). The purpose of this study was to investigate whether CIDP patients show autoreactive T cell responses against NF155 and NF186 and secondly whether CIDP and its clinical variants differ in their T cell response against NF155, NF186 as well as against the myelin epitopes P0 180–199 and MBP 82–100.

## Materials and Methods

### Standard Protocol Approvals, Registrations, and Patient Consent

The study was approved by the ethical committee of Charité University Medicine Berlin. All patients were recruited in the outpatient clinic of the Charité Department of Neurology. All patients gave their written informed consent for the study. Pseudonyms were used for the study.

### Patients

For our study, 48 patients with typical CIDP (*n* = 18), DADS (*n* = 8), MADSAM (*n* = 9), and sensory CIDP (*n* = 13) were recruited. Diagnoses were made according to the criteria of the European Federation of Neurological Societies/Peripheral Nerve Society (EFNS/PNS) (13). We assessed the clinical condition of patients by Medical Research Council (MRC) (14), and the inflammatory neuropathy cause and treatment (INCAT) disability score (15). For classification, we used CIDP disease activity status (CDAS) (16), summarizing unstable active and improving status as unstable stage, stabile active status and remission status as stable stage. We used symptoms stated in case histories for analysis of clinical features. Patients had received no immunosuppressive drugs at the time of study entry or during the six previous months. Positive treatment response was defined as an improvement of two or more points on the MRC sum score in two different muscle groups, or an improvement of one point or more on the INCAT score, or an improvement of the walking distance of more than 50% compared to baseline results (6, 17). As controls, we included 19 patients with other non-immune polyneuropathies (ONs) such as idiopathic axonal polyneuropathy (*n* = 13), metabolic or toxic polyneuropathy (*n* = 1), hereditary polyneuropathy (*n* = 1) or diabetic polyneuropathy (*n* = 2), motoneuron disease (*n* = 1), ATTR amyloidosis-associated neuropathy (*n* = 1), and nine healthy controls (HCs). Clinical and experimental data of one patient have been submitted as a case report elsewhere (18). In addition, partial MBP and P0 response data from one MADSAM and one typical patient as well as six HCs have been published previously (8).

### Blood Samples

For ELISPOT, blood samples were collected using CPT tubes (BD Vacutainer, Becton, Dickinson and Company, Franklin Lakes, NJ, USA). In patients treated with intravenous immunoglobulins (IVIg), blood was obtained on the first day of IVIg-therapy before starting the infusion. Peripheral blood monocytes (PBMCs) were isolated within 3 h by density gradient centrifugation at 1,500 *g* for 20 min and diluted in CTL-Test-Medium (CTL-Europe, Bonn, Germany) at a concentration of 6 × 10^6^ cells/mL.

### ELISPOT

For ELISPOT assay, we used our established protocol (6) based on the Elispot protocol established previously (19, 20). Briefly, 96-well plates (Millipore, Billerica, MA, USA) were coated with an interferon-gamma (IFN-γ)-specific antibody (eBioscience, San Diego, CA, USA) at 4 μg/mL and left overnight in sterile PBS. After blocking with sterile PBS + 1% BSA (Sigma-Aldrich, St. Louis, MO, USA) for 60–120 min, fresh PBMCs were added in a number of 4 × 10^5^ cells/well in presence of anti-CD28 antibody which enhances the costimulatory signal (21) (eBioscience) at 2 μg/mL. Peripheral myelin antigens MBP 82–100 and P0 180–199 as well as NF155 and NF186 were added at 40 μg/mL. As a positive control, we used CEF at a concentration of 10 μg/mL. CEF is a peptide pool containing 23 MHC class 1 restricted viral antigens (22). To detect spontaneous IFN-γ secretion, we used CTL-Test-Medium (CTL, Cleveland, OH, USA). Plates were incubated at 37°C and 5% CO_2_ for 24 h. For detection, we applied mouse anti-human IFN-γ biotin antibody (eBioscience) at a concentration of 2 μg/mL and conjugated at 1:1,000 to streptavidin-horseradish-peroxidase (BioLegend, San Diego, CA, USA). Plates were developed with 3-amino-9-ethyl carbazole reagent (Sigma Aldrich, St. Louis, MO, USA). The resulting spots were detected, counted and analyzed *via* Elispot Reader (Autoimmun Diagnostika GmbH, Strassberg, Germany) and appendant iSpot 04 Software. Spot forming units (SFU) for each antigen triplicate were averaged and subtracted by average SFU of spontaneous IFN-γ secretion and then calculated for a cell quantity of 10^6^ cells. Analyzing of the data was performed in a blinded fashion.

### Antigens

Recombinant NF155 and NF186 were kindly provided by E. Meinl (MD, LMU Munich, Germany) and were described earlier (10). P0 180–199 and MBP 82–100 were provided by R. Volkmer (SD, Charité Berlin, Germany) and were described earlier (8).

### Statistics

We compared clinical baseline measurements (age, time since diagnosis, MRC, INCAT) as well as antigen-specific IFN-γ responses using Kruskal–Wallis test followed by Dunn’s multiple comparison test or Mann–Whitney *U*-test, when suitable. Fisher’s exact test was used to compare frequencies of the non-metric clinical parameters sex, response to IVIg-treatment, CDAS, tremor, sensory ataxia, neuropathic pain, asymmetric paresis, proximal paresis, drop foot, distal motoric latency, F-wave latencies, nerve conduction velocities, conduction block, and positive CSF. We calculated Spearman’s rho to analyze the relation of P0 to MBP. In order to evaluate the discrimination properties of the different antigen T cell responses for differentiating a specific type of CIPD from ON or other atypical CIPD variants, we calculated the area under the curve (AUC) and 95% confidential interval (CI) from receiver operating characteristics (ROC)-curve analyses. Good discriminative potential of an antigen response in a CIDP subtype compared to the control group was defined as an AUC > 7, which did not include 0.5 in 95% CI. To achieve higher discrimination properties, we also analyzed combinations of promising markers using multiple logistic regression with regard to the discrimination of specific disease types from ON. Statistical analyses were performed with GraphPad Prism version 6.0 (La Jolla, CA, USA). No adjustment for multiple testing was made. A *p*-value <0.05 was considered significant.

## Results

### Identification of Neurofascin- and Myelin-Derived Antigens as T Cell Targets in CIDP

**Figure 1:**
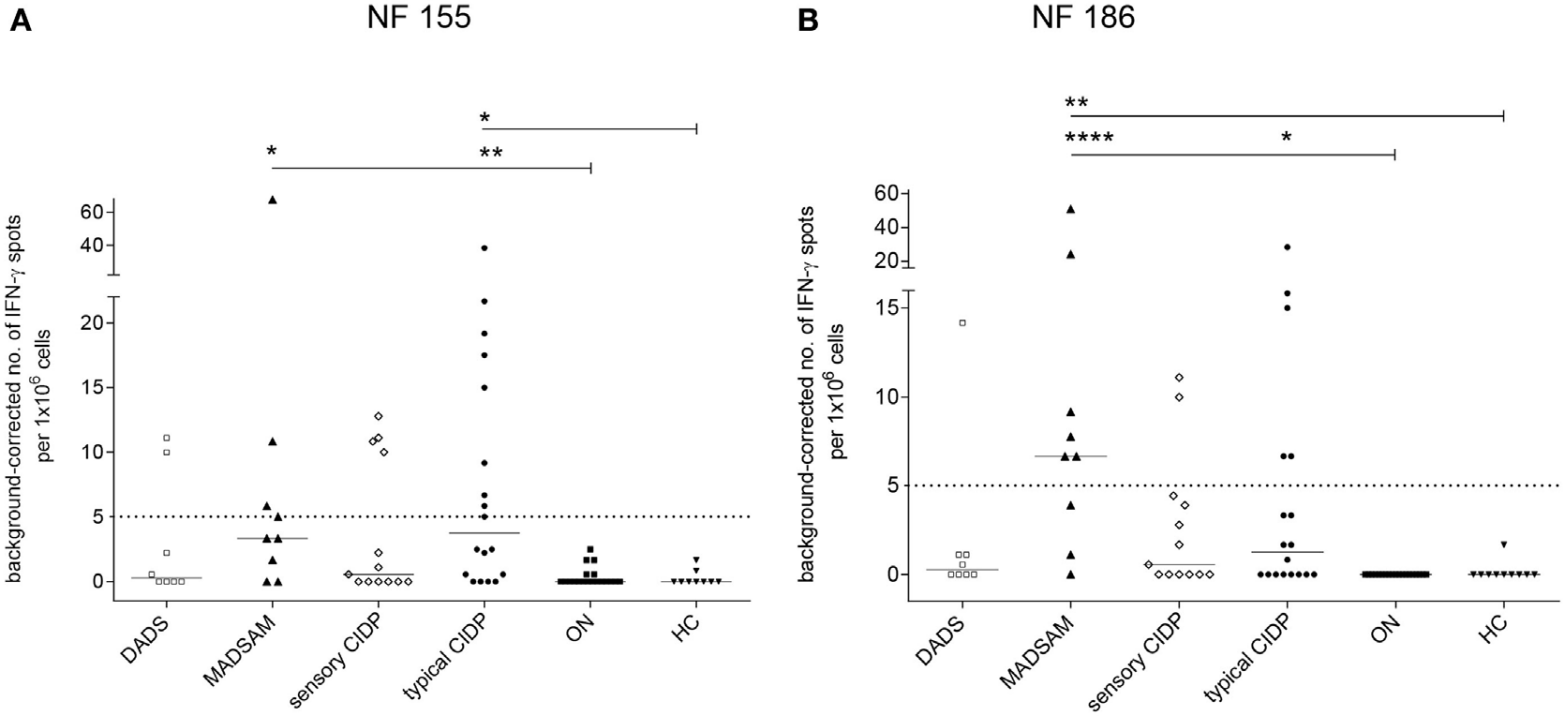
Autoreactive T cell responses against neurofascin antigens are elevated in chronic inflammatory demyelinating polyneuropathy (CIDP) subtypes compared to controls. Frequency of peripheral antigen-specific T cell responses in patients with distal acquired demyelinating polyneuropathy (DADS) (*n* = 8), multifocal acquired demyelinating sensory and motor polyneuropathy (MADSAM) (*n* = 9), sensory CIDP (*n* = 13), typical CIDP (*n* = 18), other non-immune polyneuropathy (ON) (*n* = 19), and healthy control (HC) (*n* = 9) measured by enzyme-linked immunospot assay. Lines at median. Background corrected interferon-gamma (IFN-γ) spot forming cells per 1 × 10^6^ peripheral blood monocytes against neurofascin (NF) 155 **(A)** and NF186 **(B)** (***p < 0.001, **p < 0.01, *p < 0.05; Dunn’s multiple comparisons test). **(A)** Cutoff was defined at spot forming unit (SFU) ≥ 5 with sensitivity of 44.4% and specificity of 100% based on receiver operating characteristics (ROC) analysis comparing typical CIDP and ON [area under the ROC curve (AUC) = 0.83 with 95% confidence interval of 0.7–0.96]. **(B)** Cutoff was defined at SFU ≥ 5 with sensitivity of 66.7% and specificity of 100%, based on ROC analysis comparing MADSAM and ON (AUC = 0.94 with 95% confidence interval of 0.82–1).

The NF155-specific IFN-γ response was higher in typical CIDP and MADSAM than in HC and ON (Figure 1A). Similarly, the IFN-γ response against NF186 was significantly increased in MADSAM and to a lesser extent in typical CIDP compared to HC and ON (Figure 1B). By using *post hoc* defined cutoff values (Figure 1), 9 out of 18 (50%) typical CIDP as well as 4/9 (44%) MADSAM patients responded to NF155. For NF186, 5/18 (28%) typical CIDP and 6/9 (67%) MADSAM patients exhibited positive IFN-γ responses. In contrast, DADS and sensory CIDP showed lower IFN-γ responses to NF155 and NF186. Importantly, ON and HC showed no NF-specific IFN-γ response at all. Type 1 T-helper (TH1) responses against CEF positive controls showed no differences between any of the groups (data not shown).

Serum samples from patients with immune-polyneuropathy and from controls with other polyneuropathies were analyzed for antibodies to NF155 and NF186 by ELISA. However, none of our samples showed antibody reactivity specific to NF155 or NF186 (data not shown).

**Figure 2:**
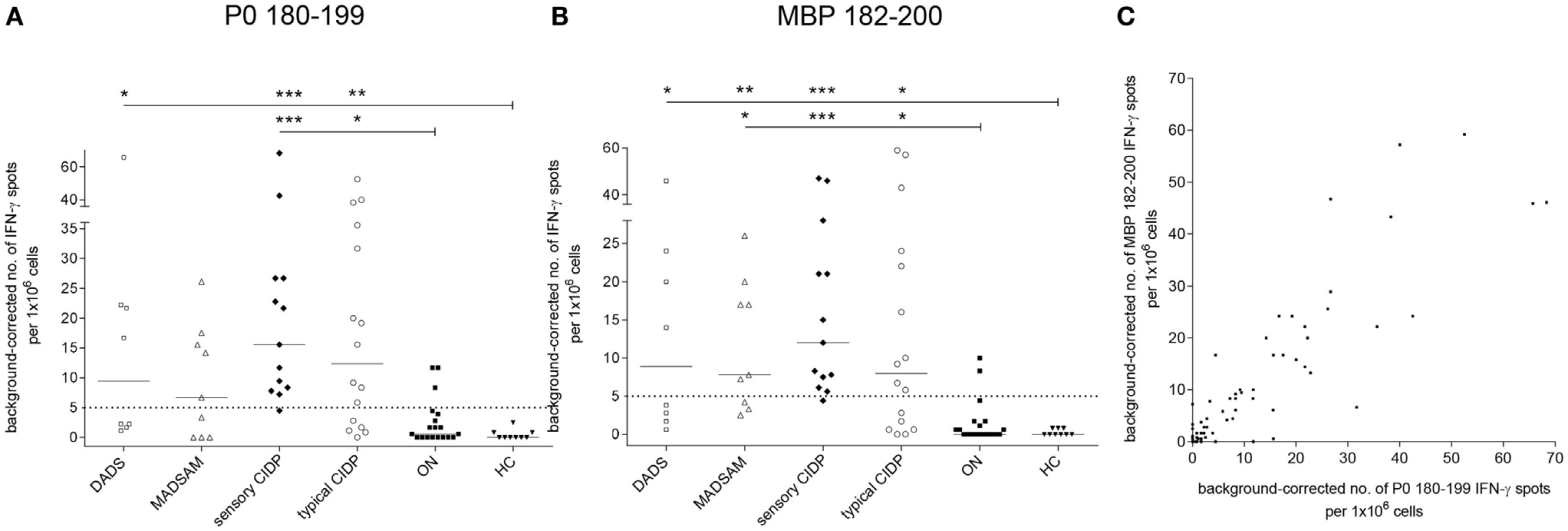
Compact myelin-specific T cell responses are elevated in chronic inflammatory demyelinating polyneuropathy (CIDP) subtypes compared to controls. Frequency of peripheral antigen-specific T cell responses in patients with demyelinating polyneuropathy (DADS) (*n* = 8), multifocal acquired demyelinating sensory and motor polyneuropathy (MADSAM) (*n* = 9), sensory CIDP (*n* = 13), typical CIDP (*n* = 16), other non-immune polyneuropathy (ON) (*n* = 19), and healthy control (HC) (*n* = 9) measured by enzyme-linked immunospot assay. Lines at median. Background corrected interferon-gamma (IFN-γ) spot forming cells per 1 × 10^6^ peripheral blood monocytes against myelin protein zero (P0) 180–199 **(A)** and myelin basic protein (MBP) 82–100 **(B)** (***p < 0.001, **p < 0.01, *p < 0.05; Dunn’s multiple comparisons test). **(A)** Cutoff was defined at spot forming unit (SFU) ≥ 5 with sensitivity of 92.3% and specificity of 84.2%, based on receiver operating characteristics (ROC) analysis comparing sensory CIDP and ON [area under the ROC curve (AUC) = 0.94 with 95% confidence interval of 0.86–1]. **(B)** Cutoff was defined at SFU ≥ 5 with sensitivity of 92.3% and specificity of 89.5% based on ROC analysis comparing sensory CIDP and ON (AUC = 0.95 with 95% confidence interval of 0.88–1). **(C)** IFN-γ reaction to MBP in correlation to P0 as measured by Spearman correlation with *r* = 0.82 (*p* < 0.001).

Sensory CIDP and typical CIDP patients showed significantly elevated P0 180–199-specific IFN-γ secretion compared to both control groups, whereas DADS patients differed significantly only from HC (Figure 2A). IFN-γ response to MBP 82–100 was elevated in all CIDP subtypes (Figure 2B). For P0 180–199, we found a positive response in 11/16 (69%) typical CIDP, in 12/13 (92%) sensory CIDP, in 5/9 (56%) MADSAM and in 4/8 (50%) DADS patients when we used *post hoc* defined cutoffs (legend Figure 2). For MBP, a positive response was detected in 10/16 (63%) typical CIDP patients, in 12/13 (92%) sensory CIDP, in 6/9 (67%) MADSAM and in 4/8 (50%) DADS patients. For ON, we found P0-specific responses in 3/19 (16%) and MBP-specific responses in 2/19 (11%), whereas investigation of HC samples revealed no antigen-specific T cell responses. Interestingly, there was a positive correlation between P0 180–199- and MBP 82–100-specific IFN-γ responses (*r* = 0.82; *p* < 0.001; Spearman *r*; Figure 2C).

In ROC analysis, the anti-NF155 response exhibited good discrimination properties when we compared typical CIDP or MADSAM to ON (Table 1A) but not to other CIDP subtypes (Table 1B). The AUC of NF186 was highest in MADSAM compared to ON (Table 1A) but also to other CIDP subtypes (Table 1B). The P0 180–199 response demonstrated good discrimination properties against ON in all but the MADSAM group, and MBP 82–100 had good discriminative potential between each CIDP subtype and ON (Table 1A). AUCs of P0 and MBP were highest in sensory CIDP compared to ON and by trend to other CIDP subtypes (Table 1). Combinations of all four markers indicated an advantage over using only the best single biomarker, but improvement was not significant (Table 1A).

**Table 1:** ROC analysis of antigen-specific T cell responses as markers for CIDP and its subtypes.

	*n* of specific type	*n* total	NF155	NF186	P0 180–199^a^	MBP 82–100^a^	*p*-Value^b^	AUC (95% CI) for combination of all 4 markers *p*-Value^c^
**A. AUC (95% CI) of specific subtype versus ON**								
Typical CID0	18	37	0.83 (0.70––0.96)	0.75 (0.62–0.89)	0.82 (0.68–0.96)	0.77 (0.61–0.93)	0.664	0.86 (0.73–1.00) (*p* = 0.362)
DADS	8	27	0.65 (0.42–0.88)	0.72 (0.52–0.93)	0.81 (0.64–0.99)	0.88 (0.76–1.00)	0.043	0.95 (0.86–1.00) (*p* = 0.147)
MADSAM	9	28	0.85 (0.67–1.00)	0.94 (0.82–1.00)	0.69 (0.44–0.94)	0.92 (0.83–1.00)	0.094	1.00 (1.00–1.00) (*p* = 0.318)
Sensory CIDP	13	32	0.67 (0.50–0.86)	0.74 (0.59–0.90)	0.94 (0.86–1.00)	0.95 (0.88–1.00)	0.011	0.99 (0.97–1.00) (*p* = 0.183)

**B. AUC (95% CI) of specific subtype versus all other subtypes**								
Typical CIDP	18	48	0.62 (0.45–0.78)	0.53 (0.35–0.7)	0.51 (0.33–0.7)	0.57 (0.37–0.76)	0.602	
DADS	8	48	0.63 (0.43–0.84)	0.64 (0.44–0.83)	0.54 (0.31–0.77)	0.55 (0.32–0.79)	0.907	
MADSAM	9	48	0.57 (0.38–0.77)	0.76 (0.59–0.93)	0.68 (0.49–0.87)	0.52 (0.34–0.7)	<0.001	
Sensory CIDP	13	48	0.6 (0.42–0.79)	0.58 (0.4–0.75)	0.65 (0.49–0.81)	0.63 (0.47–0.8)	0.504	

*Results of ROC analysis of each antigen IFN-γ response to study discriminative potential of antigenic T cell response between CIDP subtypes and ON (A) or compared to other subtypes (B)*.

*^a^Two values are missing in group of typical CIDP*.

*^b^p-Value of differences between markers*.

*^c^p-Value of combination of all four vs. best single marker*.

*CIDP, chronic inflammatory demyelinating polyneuropathy; DADS, distal acquired demyelinating polyneuropathy; MADSAM, multifocal acquired demyelinating sensory and motor polyneuropathy; MBP 82–100, myelin basic protein 82–100; NF155, neurofascin 155; NF186, neurofascin 186; ON, other non-immune polyneuropathy; P0 180–199, myelin protein zero 180–199*.

### Antigen-Specific IFN-γ Response as Supportive Diagnostic Criteria for CIDP

**Figure 3:**
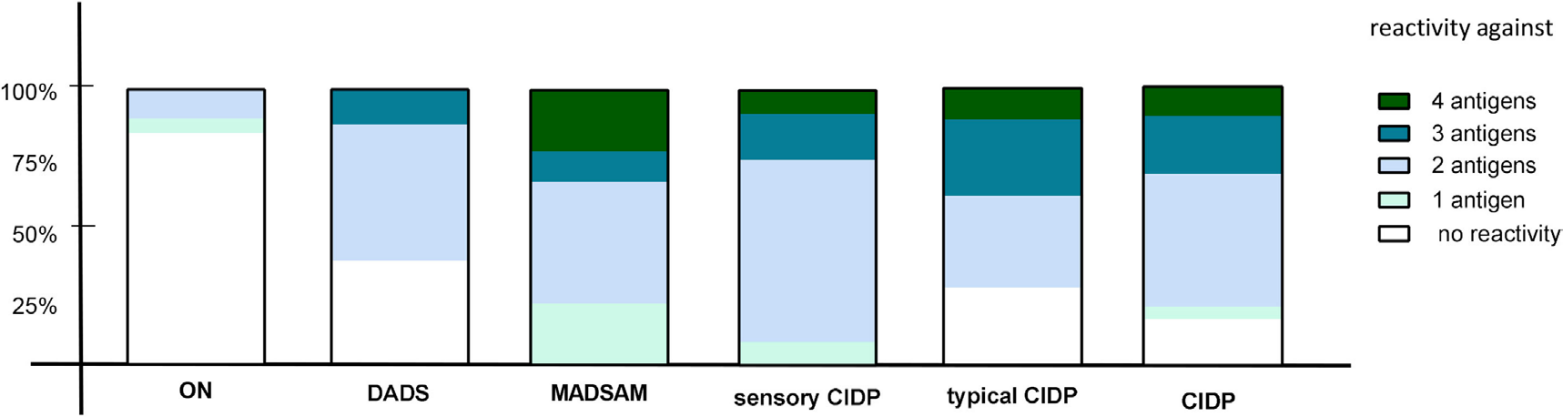
Diversity of antigenic response in chronic inflammatory demyelinating polyneuropathy (CIDP) and subtypes. The percentage of reactivity against four, three, two, one, or none of the tested antigens [neurofascin (NF) 155, NF186, P0 180–199, and MBP 82–100] is shown in other non-immune polyneuropathy (ON), demyelinating polyneuropathy (DADS), multifocal acquired demyelinating sensory and motor polyneuropathy (MADSAM), sensory, typical CIDP as well as all CIDP patients. The immune response against at least two antigens was most frequently in sensory CIDP (92.3%), MADSAM (77.7%), and typical CIDP (72.2%) compared to DADS (62.5%) and ON (10.5%).

We used cutoff values (Figure 11) to define positive responses to tested antigens. In contrast to ON, positive IFN-γ responses against two or more antigens proved highly predictive for any subtype of CIDP (positive predictive value = 0.95) and were found in 77% of CIDP patients (Figure 3). Negative IFN-γ responses to three or four antigens were observed in 23% CIDP patients (Figure 3; negative predictive value = 0.61). In contrast, only two patients (10.5%) of ON showed responses against at least two antigens; 89.5% did not. These two responding patients had been diagnosed with diabetic polyneuropathy based on clinical manifestation and nerve conduction studies. Regarding the distribution for each CIDP subtype, we found that MADSAM and sensory CIDP most often displayed widespread antigen-specific response in contrast to DADS, where the majority responded only to one or two antigens (Figure 3).

### Clinical Characteristics of Patients Stratified by Antigen-Specific T Cell Responses

Asymmetric paresis was seen in the NF186 responsive group. The MBP responsive group was male and showed a shorter disease duration compared to the MBP-unresponsive group. Similarly, patients responsive to P0 were male and younger than the P0 negative group. Looking at demyelinating parameters, we found by trend an association between the NF186 response and conduction blocks (*p* = 0.06; Fisher’s exact test). Clinical disease activity status, however, improvement upon IVIg-treatment, or clinical features such as tremor and ataxia were independent of T cell reaction specific to NF155, NF186, P0 180–199, or MBP 82–100 (Table 2). Limited antigen-specific T cell responses (response against <2 antigens) were seen in patients who were female and older than patients who showed a positive response against ≥2 antigens. Patients with limited antigen-specific response showed a lower MRC and increased INCAT score as well as more neuropathic pain and proximal paresis. F-wave latencies were less likely to be prolonged (Table 2).

**Table 2:** Clinical characteristics of ELISPOT antigen-responsive patients and patients with limited antigen-specific response.

	NF155 pos.	NF186 pos.	P0 180–199 pos.	MBP 82–100 pos.	Limited response (<2 antigen pos.)
*n* of patient (%)	19/48 (40)	14/48 (29)	32/48 (67)	32/48 (67)	11/48 (23)
Sex, male (%)	14 (74)	11 (79)	26 (81)^a^	26 (81)^a^	4 (36)^b^
Age, mean (range)	58 (27–80)	61 (53–77)	60 (27–82)^a^	60 (27–82)	73 (63–77)^b^
Time since diagnosis, median (range)	4 (0–10)	3 (0–10)	3 (0–17)	2 (0–17)^a^	5 (1–23)
Good response to IVIg-therapy, *n* (%)	16/18 (89)	11/14 (79)	21/29 (72)	23/30 (77)	9/10 (90)
Unstable disease, *n* (%)	8 (42)	8 (57)	16 (50)	17 (53)	6 (55)
MRC, mean (range)	74 (63–80)	74 (66–78)	73 (45–80)^a^	73 (45–80)	72 (62–76)^b^
INCAT, median (range)	3 (1–4)	3 (1–6)	3 (1–7)	3 (1–7)	4 (2–6)^b^
Tremor, *n* (%)	8 (42)	6 (43)	12 (38)	13 (41)	8 (73)
Sensory ataxia, *n* (%)	14 (74)	10 (71)	22 (69)	20 (63)	9 (82)
Neuropathic pain, *n* (%)	7 (37)	6 (43)	13 (41)	13 (41)	10 (91)^c^
Asymmetric paresis, *n* (%)	9 (47)	10 (71)^a^	13 (41)	14 (44)	3 (27)
Proximal paresis, *n* (%)	10 (52)	10 (71)	12 (38)^a^	13 (41)	9 (82)^b^
Drop foot, *n* (%)	13 (68)	12 (86)	18 (56)	18 (56)	10 (91)
Distal motoric latency, *n* (%)	8 (42)	5 (36)	10 (31)	9 (28)	5 (10)
F-wave latencies, *n* (%)	15 (79)	12 (86)	23 (72)	22 (69)	4 (8)^b^
Nerve conduction velocities, *n* (%)	15 (79)	8 (57)	20 (63)	20 (63)	7 (15)
Conduction block, *n* (%)	4 (21)	6 (43)	7 (22)	9 (28)	2 (4)
Positive CSF, *n* (%)	13 (68)	10 (71)	16 (50)^a^	16 (50)^a^	8 (17)

*Positive electroneurographic parameters (prolonged distal motor latency, F-wave latency, nerve conduction velocity, conduction block) were defined according electrodiagnostic criteria of EFNS (13)*.

*^a^p-Value for antigen positive patients in comparison to negative group: p < 0.05*.

*^b^p-Value for group of patients with negative reaction to three or more antigens in comparison to group with two or more positive reaction: p < 0.05*.

*^c^p-Value for group of patients with negative reaction to three or more antigens in comparison to group with two or more positive reaction: p < 0.001*.

*INCAT, inflammatory neuropathy cause and treatment; IVIg, intravenous immunoglobulin; MBP 82–100, myelin basic protein 82–100; MRC, Medical Research Council; NF155, neurofascin 155; NF186, neurofascin 186; P0 180–199, myelin protein zero 180–199*.

## Discussion

In the present study, we demonstrated specific IFN-γ T cell responses against the paranodal/nodal antigens NF155 and NF186 as well as against myelin-derived antigens MBP 82–100 and P0 180–199 in CIDP compared to ON. Positive IFN-γ responses against two or more antigens were highly predictive for any subtype of CIDP, with MADSAM and sensory CIDP showing the broadest immune response to the four tested antigens. ROC analysis indicated highest discriminative potential of NF186-specific T cell responses in MADSAM and the highest discriminative potential of P0 180–199 and MBP 82–100-specific response in sensory CIDP, suggesting the presence of cell-mediated immune responses against these antigens as a suitable biomarker for CIDP diagnosis.

The autoreactive T cell responses against NF155 and NF186 that we demonstrated here for typical CIDP and MADSAM might be of particular relevance. Recently, IgG4 antibodies to NF155 have been found in a clinically distinct subgroup of CIDP that show a younger age at onset, tremor, ataxia, CNS demyelination and a poor response to IVIg treatment which was not compatible with established EFNS classification criteria (11, 23). Based on the fact, that NF155-specific antibodies have been found in this clinically distinctive subgroup, we also correlated antigen-specific IFN-γ response with a number of clinical features (Table 2). We did not find any association between ataxia, tremor, pain, or type of paresis. In contrast to recent NF155 antibody studies (11), however, patients showing IFN-γ T cell responses against NF155 responded well to immunomodulatory treatment, which may suggest a more active T cell driven autoimmune process. In the present study, we found that positive T cell responses against NF155 and NF186 were more frequent than has been reported up to now in published antibody findings (10, 11, 23). This supports the hypothesis of an underlying T cell-mediated immune response (5, 6). Our results of NF-specific T cell reactivity further support the importance of the Ranvier node as an immunological target in CIDP. However, evidence is still lacking whether the detected antigen-specific T cell response may contribute to the disease. On the other hand, T cell response may just have evolved by tissue damage and not be pathogenic. Based on our control data of patients with non-immune neuropathy showing no or very low T cell reactivity a secondary immune response does not seem to play an important role. The elevated IFN-γ response against NF186 in MADSAM was clearly distinct from ON and other CIDP subtypes, which points to NF186 as a promising diagnostic marker to be validated in a larger multicentric study.

In the present study, we detected IFN-γ responses against P0 and MBP in 67% of our CIDP cohort. P0 is one of the major peripheral myelin proteins that functions as autoantigen in models of autoimmune peripheral neuropathy (24, 25). There is still little evidence about the role of MBP 82–100 in the pathogenesis of CIDP even though MBP has been detected as part of the myelin sheath of peripheral nerves (26). Here, we found in CIDP elevated IFN-γ responses against both P0 and MBP that show highest discriminating properties in sensory CIDP compared to ON and by trend to other subtypes. Sensory CIDP is often difficult to differentiate from non-immune polyneuropathy, since it often does not correspond with the current EFNS/PNS diagnostic criteria (13, 27, 28). P0 180–199 and MBP 82–100-specific responses may therefore be suitable candidates as diagnostic markers to support the diagnosis of sensory CIDP. Early diagnosis of CIDP and treatment initiation is essential in order to prevent irreversible axonal damage and thus disability. On the other hand, there is increasing evidence that many patients are misdiagnosed with CIDP. Recently, Allen et al. showed in a retrospective analysis of 58 patients that about 47% of all cases had been misdiagnosed with CIDP and subsequently treated for long periods without clear evidence for efficacy of treatment (27). A diagnostic marker could help to prevent misdiagnosis and reduce side effects and costs of unnecessary treatment. It would be therefore of high relevance to validate our findings in larger multicentric studies. Even though this study is comparatively large, with a total number of 48 patients, it is still too small to firmly identify T cell epitopes and correlate them with clinical features or a specific CIDP subtype. Another limitation may be the fact that the patients included were not treatment-naive and had a rather long disease duration. This may have affected our results and thus limit the predictive evidence. Despite the fact that CIDP single subtype divisions are small we still found significant higher antigen-specific T cell responses compared to controls whereas no difference could be found for the control peptide pool (CEF). Thus, our prospectively generated explorative data support a strong hypothesis. However, due to the small numbers of CIDP subtypes the present work represents a basis for validation in a greater multicenter study In addition, including other demyelinating diseases such as Guillain-Barré-Syndrom, Multifocal Motoric Neuropathy of hereditary Charcot-Marie-Tooth1 neuropathy as controls would be helpful for understanding pathomechanism as well as for developing T cell-specific responses as a biomarker of CIDP. For that, the Elispot assay provides robust, highly reproducible data. The Elispot technique can be easily used with frozen and subsequently thawed PBMCs. ELISPOT appears to be one of the fast growing applications in biomedical research such as in vaccine development (29), HIV research (30), and cancer and allergy research (31), most of them in multicenter trials. Furthermore, comparative B and T cell ELISPOT assays are useful in the process control of kidney transplant recipients (32). The great advantage of ELISPOT assay over flow cytometry is its unsurpassed sensitivity in detecting low frequency antigen-specific T cells that secrete effector molecules. A simultaneously performed T and B cell ELISPOT assay in patients with CIDP could allow a direct comparison of memory T and B cell response in the peripheral blood. Since PBMCs can be efficiently frozen without loss of function when tested in ELISPOT assay, it can be easily used to investigate suitable progression or treatment efficacy parameters.

In our cohort, eleven CIDP patients showed only a limited antigen-specific response. Looking at their clinical characteristics, we found these patients were older, had a longer disease duration, a lower MRC, and increased INCAT score with manifest long-term damages such as severe paresis and pain syndromes. Thus, the lack in autoimmune responses in this non-responder group could be attributable to a somewhat “burnt-out” disease status. We observed asymmetric paresis in the NF186 responsive group, which is compatible with the clinical definition of MADSAM patients. Patients responding to MBP and P0 were male and younger than those in the non-responsive group. This concurs with a recent clinical description of sensory CIDP (28). In contrast, there were two patients in the ON group who responded to two antigens. These patients had been diagnosed with diabetic polyneuropathy based on clinical manifestation and nerve conduction studies. However, the antigenic response in these patients may suggest an underlying immune-mediated mechanism as is being discussed with increasing frequency regarding some forms of diabetic neuropathy (33, 34).

In summary, we provide evidence for underlying auto-reactive T cell immune responses against neurofascin as well as compact myelin epitopes in CIDP and variants. P0 180–199 and MBP 82–100 IFN-γ responses were associated with sensory CIDP, whereas NF186-specific IFN-γ response was associated with MADSAM. Further multicentric studies including other inflammatory neuropathies are required to validate these promising biomarkers with reliable cut offs as useful diagnostic tools for CIDP, its subtypes, and for treatment guidance.

## Ethics Statement

The study was approved by the ethical committee of Charité University Medicine Berlin. All patients were recruited in the outpatient clinic of the Charité Department of Neurology. All patients gave their written informed consent for the study. Pseudonyms were used for the study.

## Author Contributions

J-MD: acquisition, analysis, and interpretation of data, writing of manuscript. MS: acquisition and analyses of data. CM: analyses of data and critical revision of manuscript for intellectual content. KH, patient recruitment, critical revision of manuscript for intellectual content. EM, provision of peptides, analysis, and interpretation of data. AM: critical revision of manuscript for intellectual content, conduct of study, and supervision. JK: study concept and design, patient recruitment, analysis and interpretation of data, study supervision, and writing of manuscript.

## Conflict of Interest Statement

J-MD, MS, and CM report no disclosure. KH has received speaker honoraria or refunding of conference travel costs from Abbvie, Astellas, CSL Behring, Gilead Sciences GmbH, Pfizer, ViiV Healthcare GmbH. AM reports grants from Grifols during the conduct of the study; personal fees from Grifols and Octapharma. EM received grant support from Novartis and personal compensations from Novartis, Genzyme, and Roche. JK was supported by a Rahel-Hirsch scholarship from the Charité University Medicine, Berlin. She received personal compensation for speaker fees and advisory boards outside the submitted work from Grifols, Octapharma and CSL Behring.

## Abbreviations

CIDP, chronic inflammatory demyelinating polyneuropathy; CDAS, CIDP disease activity status; DADS, distal acquired demyelinating polyneuropathy; ELISPOT, enzyme-linked immunospot; EFNS, European Federation of Neurological Societies; HC, healthy control; INCAT, inflammatory neuropathy cause and treatment; IFN-γ, interferon-gamma; IVIg, intravenous immunoglobulin; MADSAM, multifocal acquired demyelinating sensory and motor polyneuropathy; MBP/MBP 82–100, myelin basic protein 82–100; MRC, Medical Research Council; NF155, neurofascin 155; NF186, neurofascin 186; ON, other non-immune polyneuropathy; P0/P0 180–199, myelin protein zero 180–199; PBMCs, peripheral blood mononuclear cells.
